# Palliative bypass surgery for patients with advanced pancreatic adenocarcinoma: experience from a tertiary center

**DOI:** 10.1186/s12957-020-01828-5

**Published:** 2020-04-01

**Authors:** Niv Pencovich, Lior Orbach, Yonatan Lessing, Amit Elazar, Sophie Barnes, Phillip Berman, Arye Blachar, Ido Nachmany, Boaz Sagie

**Affiliations:** 1 Department of General Surgery B, Division of Surgery, Tel-Aviv Sourasky Medical Center, 6 Weizmann St, 64239 Tel Aviv, Israel; 2 Department of Radiology, Tel-Aviv Sourasky Medical Center, Tel-Aviv, Israel

**Keywords:** Palliation, Gastrojejunostomy, Hepaticojejunostomy, Bypass surgery

## Abstract

**Background:**

As advances in oncological treatment continue to prolong the survival of patients with non-resectable pancreatic ductal adenocarcinoma (PDAC), decision-making regarding palliative surgical bypass in patients with a heavy disease burden turns challenging. Here we present the results of a pancreatic surgery referral center.

**Methods:**

Patients that underwent palliative gastrojejunostomy and/or hepaticojejunostomy for advanced, non-resectable PDAC between January 2010 and November 2018 were retrospectively assessed. All patients were taken to a purely palliative surgery with no curative intent. The postoperative course as well as short and long-term outcomes was evaluated in relation to preoperative parameters.

**Results:**

Forty-two patients (19 females) underwent palliative bypass. Thirty-one underwent only gastrojejunostomy (22 laparoscopic) and 11 underwent both gastrojejunostomy and hepaticojejunostomy (all by an open approach). Although 34 patients (80.9%) were able to return temporarily to oral intake during the index admission, 15 (35.7%) suffered from a major postoperative complication. Seven patients (16.6%) died from surgery and another seven within the following month. Nine patients (21.4%) never left the hospital following the surgery. Mean length of hospital stay was 18 ± 17 days (range 3–88 days). Mean overall survival was 172.8 ± 179.2 and median survival was 94.5 days. Age, preoperative hypoalbuminemia, sarcopenia, and disseminated disease were associated with palliation failure, defined as inability to regain oral intake, leave the hospital, or early mortality.

**Conclusions:**

Although palliative gastrojejunostomy and hepaticojejunostomy may be beneficial for specific patients, severe postoperative morbidity and high mortality rates are still common. Patient selection remains crucial for achieving acceptable outcomes.

## Introduction

Pancreatic adenocarcinoma (PDAC) is a leading cause of cancer related mortality worldwide [1]. Although oncological resection offers the best chance for prolonged survival and may even achieve cure, approximately 80% of patients are diagnosed with inoperable cancer at presentation due to locally advanced or metastatic disease [1, 2]. Traditionally, patients with locally advanced PDAC undergoing treatment with chemotherapy and radiation had a median survival of 10–12 months [3, 4]. Recent trials, however, have demonstrated that new chemo-radiotherapy regimens may substantially increase survival time and even convert unresectable tumors to resectable tumors, in a small proportion of patients [5, 6]. In patients with metastatic disease, in which median survival of 5 to 8 months was generally the rule, [1, 7] modern protocols, such as FOLFIRINOX, have significantly improved survival to a median of over 11 months [8] and are now the first-line treatment for these patients [8]. As the longevity of patients who are not suitable for curative resection improves, the role of surgical palliation in providing a durable quality of life and allowing continuous administration of systemic therapy becomes paramount [9].

Approximately 70% of patients with PDAC present with obstructive jaundice, and gastric outlet obstruction (GOO) occur in 25%, usually later in the course of disease, due to direct tumor invasion [10]. With the advances in systemic therapy, these rates are expected to rise, placing even further weight on palliative procedures in the management of PDAC [11]. Surgical palliation for obstructive jaundice and GOO that was performed during an operation with a curative intent in which an unanticipated advanced disease was discovered and was shown to have a relatively low failure rate [12]. However, in patients with an already proven advanced disease, who develop tumor-related obstructive symptoms, surgical bypass was traditionally shown to be associated with a high rate of major complications frequently precluding return to systemic therapy, prolonged length of hospital stay, and substantial perioperative mortality [9]. Nevertheless, in carefully selected patients, long-term alleviation of obstructive symptoms may be achieved, leading to substantial improvement of quality of life and allowing ongoing administration of systemic therapy [12, 13]. Patient selection is crucial in palliative surgery, as patients are clearly dichotomized into those who derive great benefits, and others in which suffering is enhanced and mortality hastened. In spite of studies performed during the last two decades, patient selection for palliative surgeries remains a challenge [14, 15]. Here we present our experience in palliative bypass surgeries for patients with advanced PDAC and aim to define exclusive criteria in the current era.

## Methods

### Patients

Patients that underwent palliative gastrojejunostomy and hepaticojejunostomy for pancreatic adenocarcinoma between January 2010 and January 2018 were included. Patients’ demographics, comorbidities, preoperative and surgery parameters and postoperative course including complications, return to oral food intake, length of stay, return to systemic chemotherapy, and long-term outcomes, were retrospectively obtained from patient files. Postoperative mortality was defined as any death within 30 days from surgery or mortality in the same hospital stay. Postoperative morbidity was graded according to the Clavien-Dindo (CD) classification [16].

“Palliation failure” was defined as either inability to regain oral intake, failure to discharge, or 60 days postoperative mortality.

### Sarcopenia assessment

We measured the cross-sectional area of muscles at the L3 vertebral level on computed tomography (CT) imaging. The preoperative scans and DICOM data were imported into the IntelliSpace Portal (version 9.0, Philips Medical Systems), and an area measurement was acquired from a selected axial slice using a “freehand contour” tool. The area was divided by height squared to calculate skeletal muscle index (SMI). Cutoffs for sarcopenia were based on a CT-based study for patients with malignancies (L3 SMI ≤ 38.5 cm^2^/m^2^ for women and L3 SMI ≤ 52.4 cm^2^/m^2^ for men) [17].

### Statistical analysis

Statistical analysis was performed using the IBM SPSS statistics data editor. Continuous data is expressed as median values with the corresponding standard deviation. Student’s *t* test was used for continuous data, and chi-squared test was used for categorical data. The COX regression model with stepwise forward selection was used to determine predictors of OS with results presented as hazard ratios (HRs) and 95% confidence intervals (95% CI). Binary logistic regression was used to assess predictors of palliation failure to regain oral intake, occurrence of major complications, perioperative mortality, and receiving chemotherapy. Factors included in the multivariate model were selected based on clinical relevance. Cumulative survival curves were plotted using the Kaplan-Meier method and statistically compared using the log-rank test.

## Results

### Patient population

Between January 2010 and January 2018, 42 patients (19 females) underwent palliative gastrojejunostomy and/or hepaticojejunostomy for advanced PDAC, proven either by imaging or by surgical exploration performed prior to the index palliative surgery. All patients suffered from obstructive symptoms attributed to the local effect of the primary lesion. Twenty-one patients had metastatic disease (Table 1). Of these, 14 had peritoneal spread, 10 had liver metastasis, and 4 had both liver and other intra-abdominal metastases. Two patients had extra-abdominal metastases (Table 1). Twenty-nine patients (50%) had locally advanced disease only (Table 1). The mean age at surgery was 70.5 ± 11.44, and the average body mass index (BMI) was 23.97 ± 5.23 kg/m^2^. The most frequent comorbidities were hypertension (HTN) (57.1%), diabetes mellitus (DM) (40.4%), hyperlipidemia (40.4%), and a history of ischemic heart disease (IHD) (21.4%) (Table 1). Ten patients (23.8%) were active smokers. Ten patients (23.8%) underwent previous abdominal operations. Thirteen patients (30.9%) received chemotherapy before the palliative surgery. Fifteen patients (35.7%) had prior biliary drainage, either by percutaneous transhepatic drain (PTD) or by endoscopic retrograde cholangiopancreatography (ERCP) (Table 1). Relevant patient blood indices are listed in Table 1.

**Table 1:** Patient characteristics and preoperative data

	*n* = 42
Gender, m/f (ratio)	23/19 (1.21)
Age (average ± SD)	70.5 ± 11.44
BMI (average ± SD)	23.97 ± 5.23
Metastatic disease, *n* (%)	21 (50)
Intra-abdominal metastasis	14 (33.3)
Liver metastasis	10 (23.8)
Extra-abdominal metastasis	2 (4.7)
Locally advanced disease only, *n* (%)	21 (50)
Comorbidities, *n* (%)	
HTN	24 (57.14)
DM	17 (40.4)
HPL	17 (40.4)
IHD	9 (21.4)
CHF	2 (4.7)
CKD	3 (7.14)
CVA	4 (9.4)
BPH	3 (7.14)
COPD	1 (2.35)
Smoking	10 (23.8)
Obesity	3 (7.14)
Past abdominal surgery, *n* (%)	10 (23.8)
Preop chemotherapy, *n* (%)	13 (30.9)
Preop PTD, n (%)	4 (9.4)
Preop ERCP, n (%)	11 (26.2)
Preop surgical exploration, n (%)	5 (11.9)
Preop albumin (mean ± SD)	34.6 ± 4.94
Preop albumin< 30, n (%)	6 (14.28)
Preop bilirubin> 1, n (%)	14 (33.3)

*BMI* body mass index, *HTN* hypertension, *DM* diabetes mellitus, *IHD* ischemic heart disease, *CHF* congestive heart failure, *CKD* chronic kidney disease, *HPL* hyperlipidemia, *BPH* benign prostate hypertrophy, *CVA* cerebrovascular accident, *COPD* chronic obstructive pulmonary disease, *PTD* percutaneous transhepatic drainage, *ERCP* endoscopic retrograde cholangiopancreatography

### Operative parameters

Thirty-one patients (73.8%) underwent gastrojejunostomy only, and 11 (26.1%) underwent a “double-bypass”. Twenty-two (70.9%) of the 31 gastrojejunostomies were performed laparoscopically. All double bypasses were performed by an open approach. Overall surgery time was 205 ± 78 min, open gastrojejunostomy time was 225 ± 71 min, laparoscopic gastrojejunostomy time was 162 ± 53 min, and double-bypass took 270 ± 76 min (Table 2). No conversions to an open approach were needed. No blood units were administered during surgeries (Table 2).

**Table 2:** Surgery and postoperative course

	*n* = 42
Laparoscopy/open (ratio)	22/20 (1.1)
GJ only, *n* (%)	31 (73.8)
Double bypass, *n* (%)	11 (26.1)
Overall surgery time, min (mean ± SD)	205 ± 78
Open gastro-jej time, min (mean ± SD)	225 ± 71
Lap gastro-jej time, min (mean ± SD)	162 ± 53
Double bypass surgery time, min (mean ± SD)	270 ± 76
Patients requiring blood units in op, *n* (%)	–
Postoperative complications, *n* (%)	
Clavien-dindo ≥ 3	15 (35.7)
Leak of anastomosis	4 (9.5)
Bleeding	4 (9.5)
DVT	2 (4.76)
Reoperation within 30 days	6 (14.2)
Return to oral intake during post-op, *n* (%)	34 (80.9)
Length of stay, days (mean ± SD, range)	18.3 ± 22.2, 2–108
30 days mortality, *n* (%)	7 (16.6)
60 days mortality, *n* (%)	14 (33.3)
Returning to chemotherapy, *n* (%)	18 (42.8)
Revisional palliative surgery, *n* (%)	3 (7.14)
Palliation failure	17 (40.5)
Overall survival (mean ± SD)	172.8 ± 179.2
Median survival	94.5

*DVT* deep vein thrombosis, *Lap* laparoscopic, *GJ* gastrojejunostomy

### Postoperative course

Fifteen patients (36%) suffered from major postoperative complications (Clavien-Dindo ≥ 3) (Table 2). Leaking from anastomosis occurred in 4 patients (9.4%), and bleeding requiring intervention, occurred in other 4 patients, one of which died within 30 days from surgery (Table 2). Two patients with anastomotic leak died within 30 days from surgery. The other two patients who leaked died within 62 and 128 days. Reoperation was required in 6 patients (14.2%) and three of them died shortly after reoperation within 30 days from the index operation. Mean length of hospital stay was 18.3 ± 22.2 days, five patients stayed longer than one month following surgery, and one stayed for 87 days until discharged to a nursing facility. Seven patients (16.6%) died within 30 days from surgery and another seven within the following month. Nine patients (21.4%) never left the hospital following surgery. Thirty-four patients (80.9%) were able to return to oral intake during the index admission, although many of this was soon interrupted by further complications. Only 18 patients (42.8%) were able to receive systemic therapy following surgery (Table 2). Mean overall survival was 172.8 ± 179.2, and median survival was 94.5 days (Table 2).

### Factors associated with failure of palliation and mortality

**Fig. 1:**
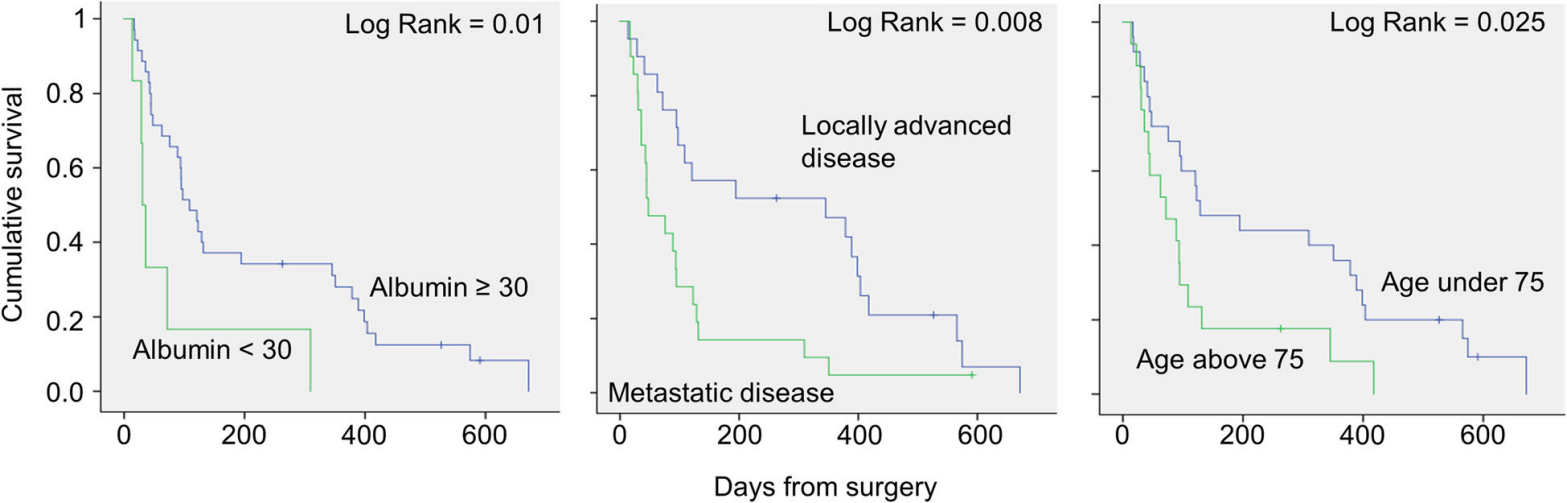
Survival curves of patients that underwent palliative bypass from non-resectable for pancreatic adenocarcinoma demonstrating decreased survival in those with preoperative albumin below 30 g/dl (left), metastatic disease, and age over 75 years

Multivariate analysis demonstrated that failure of palliation, as previously defined as inability to regain oral intake, leave the hospital, or early mortality, was significantly associated with preoperative decreased albumin level (*p* = 0.034) and increased age (*p* = 0.027). Multivariate survival analysis using backward stepwise selection revealed that early postoperative mortality was associated with preoperative sarcopenia, metastatic disease, and preoperative albumin level (Table 3). Trends toward association with older age (over 75), preoperative chemotherapy, and application of double bypass were found but did not reach statistical significance (Table 3). Log-rank test exhibited significantly worse survival for patients with albumin level below 30 g/L (*p* = 0.01), metastatic disease (*p* = 0.008), and those above the gae of 75 (*p* = 0.025) (Fig. 1).

**Table 3:** Multivariate analysis of factors associated with early mortality

	Hazard ratio	95.0% CI hazard ratio		
		Lower	Upper	*P*
Sarcopenia–L3 SMI	0.954	0.912	0.999	0.046
Metastatic disease	0.425	0.193	0.938	0.034
Preoperative albumin	0.895	0.828	0.968	0.006
Age	1.032	0.996	1.069	0.082
Preoperative chemotherapy	2.034	0.915	4.524	0.082
Double bypass	2.196	0.961	5.021	0.062

## Discussion

Palliative surgery for PDAC may be considered in three subgroups of patients with obstructive symptoms: (1) newly diagnosed patients, presenting with a non-resectable disease, (2) patients that were taken to surgical exploration with a curative intent in which a non-resectable disease was unexpectedly found, and (3) patients with long standing disease presenting either with recurrence following resection or following/during systemic therapy. In the first two scenarios, the surgeon usually follows a relatively simple decision-making algorithm. In the first scenario, the newly diagnosed patient has just taken the first steps in the course of disease and usually shares a common motivation with the treating surgeon to quickly and effectively alleviate the obstruction striving to initiate systemic treatment as soon as possible. Performance status is preserved, and discussion regarding palliative surgery focuses on the “how” and not the “if.” Deciding between surgical and endoscopic alternatives is the key here, taking into account both durability and periproceadural potential risk. In the second scenario, the ability to withstand surgery is rarely ever an issue, as these patients were originally intended for a significantly larger operation. The decision to perform a surgical bypass relies on the existence of preoperative obstructive symptoms and is usually made prior to surgery. In both scenarios, favorable outcome of palliative surgery is common due to a usually preserved patient performance status. On the other hand, the third group of patients poses a serious dilemma. These patients are severely affected by the burden of disease as well as previous interventions and treatments, often presented with depleted physical and psychological reserves. Here, decision-making includes the “if”, as well as the “how”, taking into consideration issues of patient selection, procedure planning, and timing of intervention. Other therapeutic means including endoscopy, chemo-radiotherapy, and interventional radiology, which may provide effective but usually short-term palliation, and should also be discussed in relation to the expected survival. The patient’s and family’s end of life preferences may considerably influence the decisions. Previous series have demonstrated poor results in this subgroup of patients [18, 19]. Indeed, the morbidity and mortality of bypass procedures in our patients were shown to be very high, with 30% of patients succumbing after 60 days from surgery. As a reference, the mortality of pancreaticoduodenectomy by the same team over the same time frame was less than 5% [20]. Failure to achieve main goals such as return to oral intake, hospital discharge, and short-term survival occurred in over 40% of our patients. This was associated with preoperative hypoalbuminemia and increased age. Early postoperative mortality, specifically, was associated with preoperative sarcopenia, metastatic disease, and hypoalbuminemia. Since the association between preoperative metabolic reserve, manifested among others by sarcopenia and decreased albumin levels, and heavily influenced by age and disease burden is well known by the common surgeon, one can contemplate why patients in this condition were selected for surgery. It is our belief that various biases influenced the surgeons, including, among others, the patient’s and family’s motivation to dedicatedly maintaining a proactive approach, mainly due to the short-time interval from diagnosis to these end of life decisions. This may also be influenced by cultural and religious factors. Further studies are warranted in order to identify factors that drive surgeons to operate in spite of clearly poor preoperative prognostic factors.

One study limitation is its seemingly small cohort size. However, due to the aggressive nature of advanced PDAC, published series on palliative surgical bypass, in patients with already known advanced disease, are relatively narrow as many of these patients are not fit for surgery due to previously mentioned reasons. The retrospective nature of the study is also a limitation. However, due to ethical issues, performing a controlled clinical trial to determine the effectiveness of surgical palliation would be very complex.

## Conclusions

Surgical palliation for obstructive symptoms in pancreatic adenocarcinoma can be performed in selected patients with acceptable morbidity and mortality. The association between reduced preoperative metabolic reserve, manifested by sarcopenia, decreased albumin levels, old age and disease burden, and poor outcome, is familiar to surgeons and adhering to strict patient selection remains the main challenge. Establishing guidelines or expert consensus in the field of surgical palliation should be considered in order to improve preoperative assessment and avoiding futile surgical intervention.

## Data Availability

The datasets used and/or analyzed during the current study are available from the corresponding author on reasonable request.

## Abbreviations

BMI: Body mass index
BPH: Benign prostate hypertrophy
CD: Clavien-Dindo
CHF: Congestive heart failure
COPD: Chronic obstructive pulmonary disease
CRF: Chronic renal failure
CT: Computed tomography
CVA: Cerebrovascular accident
DM: Diabetes mellitus
DVT: Deep vein thrombosis
ERCP: Endoscopic retrograde cholangiopancreatography
GJ: Gastrojejunostomy
GOO: Gastric outlet obstruction
HPL: Hyperlipidemia
HR: Hazard ratio
HTN: Hypertension
IHD: Ischemic heart disease
PDAC: Pancreatic ductal adenocarcinoma
PTD: Percutaneous transhepatic drain
SMI: Skeletal muscle index
